# Acute abdomen caused by rupture of a torted intra-abdominal testicular mass: Case report

**DOI:** 10.1016/j.ijscr.2020.02.067

**Published:** 2020-03-09

**Authors:** Ahmed Ghobashy, Doaa Hasan, Ahmed AbdElsalam, Aboubakr Ahmed, Ahmed Arafat, Mahmoud Tarek, Moutaz Ragab

**Affiliations:** a Department of General Surgery, KasrAlainy Faculty of Medicine, Cairo University, Egypt; b General Surgery Resident, Imbaba General Hospital, Cairo, Egypt; c Department of Surgery, Pediatric Surgery Unit, Cairo University Specialized Pediatric Hospital (CUSPH), Faculty of Medicine, Cairo University, Cairo, Egypt

**Keywords:** Cryptorchidism, Germ cell tumor, Testicular cancer, Undescended testis, Intra-abdominal mass

## Abstract

**Introduction:**

Cryptorchidism is defined as the absence of one or both testicles from the scrotum, which is considered one of the most common birth defects of male genitalia. Mostly the undescended testes are in the inguinal region, while the intra-abdominal location accounts for only 10 % of the cases. The potential risk of malignant transformation in an undescended testis is approximately 2.5–8 times higher than in scrotal position. Torsion of the cryptorchid testis is a very rare cause of acute abdominal pain with few cases published in literature.

**Case presentation:**

A 44-year-old male patient presented to the emergency room with acute abdominal pain. General examination revealed relevant tachycardia. Upon abdominal examination there was tenderness and rebound tenderness all over the abdomen, more evident in the lower abdomen. Interestingly, routine examination of the inguino-scrotal region revealed empty right hemi-scrotum despite the patient not being aware. Routine blood tests showed marked anemia Hb: 7.4 g/dl. Urgent pelvi-abdominal U/S showed moderate free intra-abdominal collection in addition to a pelvic mass. CT of the abdomen showed a well-defined heterogenous hypodense mass measuring about 8.5 × 5.5 cm in the pelvis. Urgent exploratory laparotomy was done, and a mass was found to be torted and ruptured. Excision was done and histo-pathological examination revealed testicular mixed germ cell tumor.

**Conclusion:**

Undescended testis is associated with an increased risk of infertility, testicular cancer and torsion. In any male with undescended testis and intra-abdominal mass; the risk of intra-abdominal testicular tumor should be considered.

## Highlights

• Scrotal examination is an integral part of the abdominal examination.
• Intra-abdominal location of undescended testis accounts for about 10 % of the cases.
• Intra-abdominal location harbors a higher risk for malignancy.
• Torsion of the cryptorchid testis is a very rare cause of acute abdominal pain.

## 1 Introduction

Cryptorchidism is defined as the absence of one or both testicles from the scrotum, which is considered one of the most common birth defects of male genitalia [1]. The estimated incidence of this disorder ranges between 1 % and 4% in full-term newborns and up to 30 % in preterm males [2]. Mostly the undescended testes are in the inguinal region, while the intra-abdominal location accounts for only 10 % of the cases [3].

The potential risk of malignant transformation in an undescended testis is approximately 2.5–8 times higher than in normal population [4]. Moreover, the intra-abdominal location has a higher risk of malignancy 5 times more than all undescended testes [5]. Torsion of the cryptorchid testis, whether inguinal or intra-abdominal, is a very rare cause of acute abdominal pain [6] with few cases published in literature.

Here we present a case of intra-abdominal testicular mass complicated by torsion, rupture and internal hemorrhage mimicking acute abdomen. This work has been reported in line with the SCARE criteria [7].

## 2 Case presentation

A 44-year-old male patient who is not known to be diabetic or hypertensive was presented to the emergency room with sudden diffuse abdominal pain for 8 h with no history of trauma. He experienced intermittent attacks of vague lower abdominal pain lasting for few hours with spontaneous relief over the last 2 days. The patient underwent open appendectomy 2 years ago; the patient is married with 3 offspring’s, the youngest of which is 12 years old.

General examination revealed relevant tachycardia (pulse116/min), normal blood pressure was 118/76 mmHg and the respiratory rate was 14 breaths / min. Oxygen saturation on room air was 99 %, and he was afebrile.

Upon abdominal examination there was tenderness and rebound tenderness all over the abdomen, more evident in the lower abdomen. Interestingly, routine examination of the inguino-scrotal region revealed empty right hemi-scrotum despite the patient not being aware about this. Routine blood tests showed high white blood cells count = 20,000 10*3 /mm and marked anemia Hb: 7.4 g/dl.

**Fig. 1:**
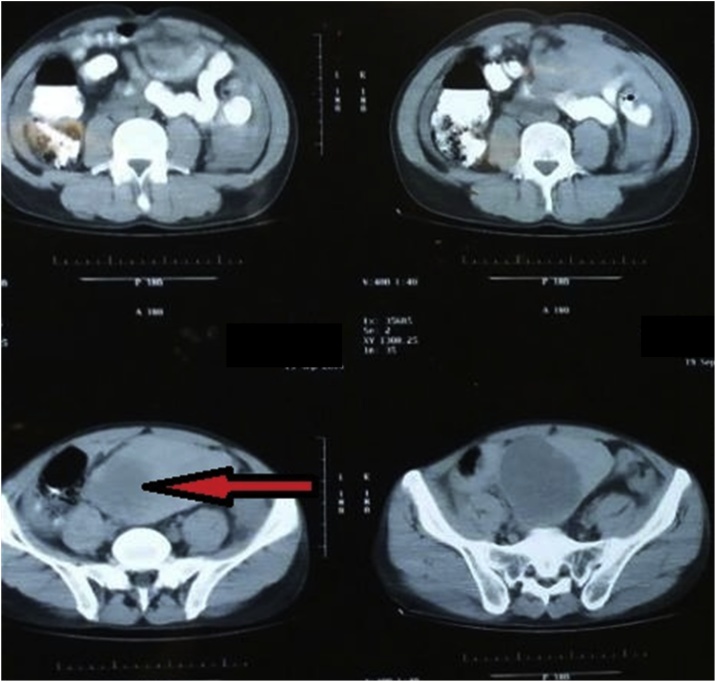
Showing mass (arrow).

Urgent pelvi-abdominal U/S showed moderate free intra-abdominal collection in addition to a pelvic mass. Computed tomography of the abdomen (Fig. 1) showed a well-defined heterogenous hypodense mass measuring about 8.5 × 5.5 cm in the pelvis.

**Fig. 2:**
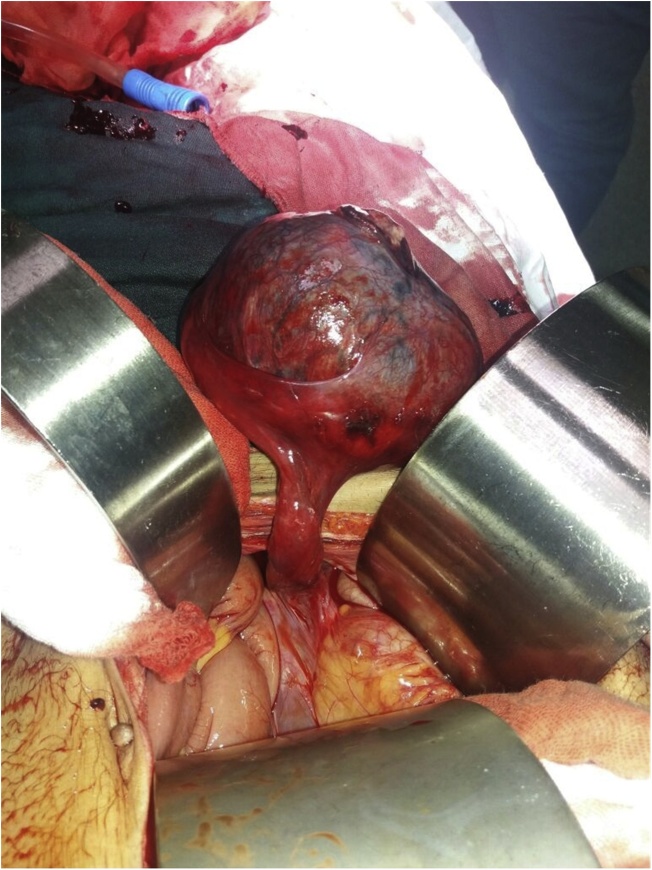
Intra-operative view.

**Fig. 3:**
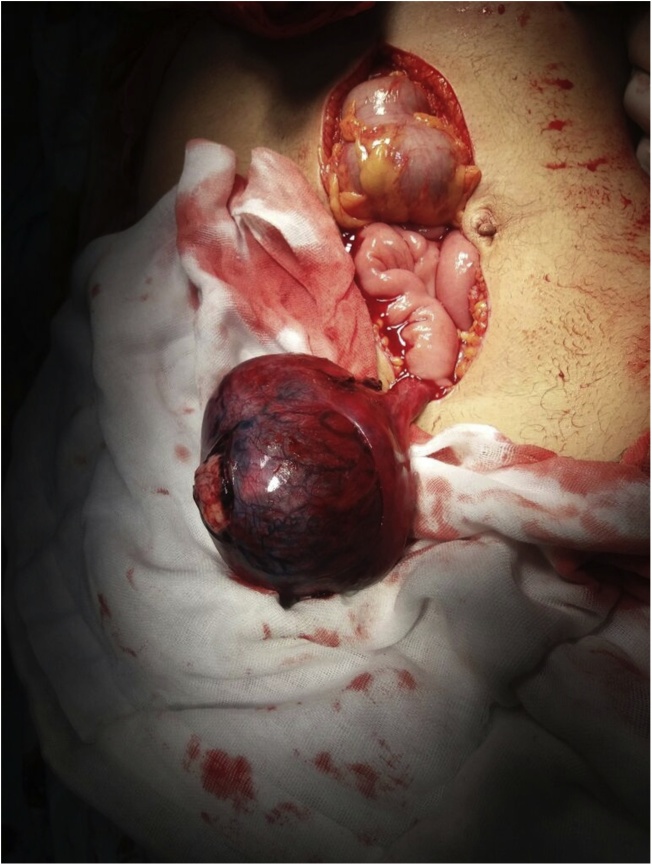
Intra-operative view.

A decision was made to proceed for urgent exploratory laparotomy owing to the drop of hemoglobin level. Initial resuscitation was performed and through a midline incision we found a rounded gangrenous perforated mass in the pelvis, attached to the lateral pelvic wall on the right side by a narrow band containing blood vessels and cord-like structure, as shown in (Figs. 2 & 3 ).

This mass was found to be torted around its band about 180 degrees counterclockwise. The gangrenous mass was excised through ligation of its vascularized pedicle flush with the lateral pelvic wall, formal abdominal exploration was done, and no evidence of suspicious lymph nodes or other masses was found. The specimen was sent for histopathological examination.

**Fig. 4:**
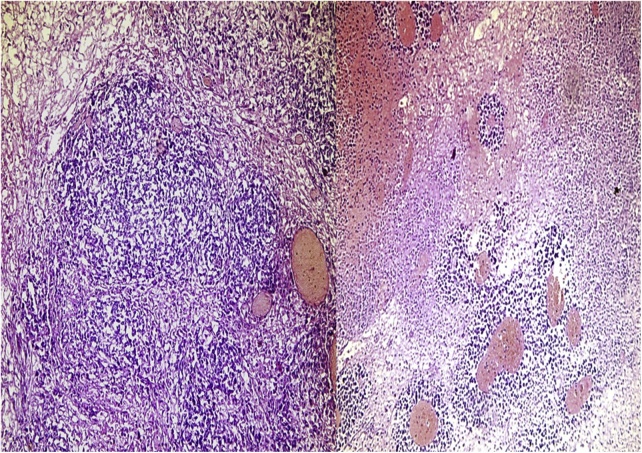
Microscopic view showing malignant cells.

Pathological and Histological examination confirmed that the mass was a necrotic hemorrhagic testicular tissue with wide areas of infarctions. Unfortunately, the pathology revealed testicular mixed germ cell tumor (Fig. 4).

The patient had an uneventful postoperative course and was discharged on the fifth hospital day to complete his course of treatment with the oncology department in our institute.

## 3 Discussion

Malignant transformation in an undescended testis usually peaks at the third or fourth decade of life, where pure seminoma represents more than 90 % of the histopathological features of undescended testicular tumors in adulthood [8]. Sub-fertility, Infertility, testicular torsion are other complications that might be associated with Cryptorchidism [9].

The Nordic group made a consensus that orchidopexy must be performed prior to one year of age for maximum preservation of potential future fertility. If the condition is diagnosed later in life, surgery should be performed as early as possible [10]. However, early orchiopexy has not been proven to reduce the potential risk of testicular cancer, yet early detection has become much more relevant even by self-examination [9].

There are many studies linking cryptorchidism to cancer. However, there are no long series of intra-abdominal testicular tumors, and most of the cases are in the form of case reports.

Abu-Zaid et al. reported a 25-year-old male with left undescended testicle presenting with a rapidly growing mass in the left inguinal region. Tru-cut biopsy revealed mixed germ cell tumor [11]. Sert et al. reported a 32 year old fertile male presented to the emergency room with vital instability and acute right lower abdominal pain, he had an empty right hemi-scrotum, exploration revealed intra-abdominal testicular tumor which was excised and the pathology turned out to be mixed germ cell tumor [12].

In our case, the right testis was intra-abdominal; the patient was married with 3 children and did not seek medical advice before, about having only one testicle. Our decision to go for urgent exploration was due to the vital instability of the patient owing to the ongoing internal bleeding, which is a very rare presentation.

Regarding the pathology in our case, it is also rare to encounter mixed germ cell testicular tumor as most cases reported in literature were pure seminoma. To the best of our knowledge this is the third reported case of intra-abdominal mixed germ cell tumor on top of a neglected undescended testis.

## 4 Conclusion

Undescended testis is associated with an increased risk of infertility, testicular cancer and torsion; therefore, it is mandatory to increase awareness of this condition among the primary care physicians. The scrotal examination is an integral part of the abdominal examination.

In any male with undescended testis and intra-abdominal mass; the risk of intra-abdominal testicular tumor should be considered.

## Declaration of Competing Interest

None.

## Sources of funding

No Funding.

## Ethical approval

Ethical approval obtained from Cairo University Ethical Committee.

## Consent

Written informed consent was obtained from the patient for publication of this case report and accompanying images. A copy of the written consent is available for review by the Editor-in-Chief of this journal on request.

## Author contribution

Ahmed Ghobashy: Main operator – reviewing the manuscript.

Doaa Hasan: Data curation.

Ahmed AbdElsalam: Conceptualization - Critical revision of the manuscript.

Aboubakr Ahmed: Data curation - Drafting the manuscript.

Ahmed Arafat: Drafting manuscript.

Mahmoud Tarek: Data collection.

Moutaz Ragab: Drafting the manuscript.

## Registration of research studies

Not applicable.

## Guarantor

Moutaz Ragab.

## Provenance and peer review

Not commissioned, externally peer-reviewed.

## References

[1] Wood H.M., Elder J.S. (2009). Cryptorchidism and testicular cancer: separating fact from fiction. J. Urol..

[2] Sijstermans K., Hack W.W., Meijer R.W., Van der Voort-Doedens L.M. (2008). The frequency of undescended testis from birth to adulthood: a review. Int. J. Androl..

[3] Nickalis O.J., Tan C.L., Thian Y.L. (2015). A tored ruptured intra-abdominal testicular seminoma presenting as an acute abdomen. J. Radiol. Case Rep..

[4] Ashley R.A., Barthold J.S., Kolon T.F. (2010). Cryptorchidism: pathogenesis, diagnosis, treatment and prognosis. Urol. Clin. North Am..

[5] Nader A.M., Moaath A.M., Jamal A.K., Mujalli M.M. (2009). Giant intra-abdominal seminoma. Saudi Med. J..

[6] Chua W., Engledow A., Raptis D., Obichere A. (2007). Torted intra-abdominal testicular tumour mimicking an appendix mass. Gd. Rounds.

[7] Agha R.A., Borrelli M.R., Farwana R., Koshy K., Fowler A., Orgill D.P., For the SCARE Group (2018). The SCARE 2018 statement: updating consensus Surgical CAse REport (SCARE) guidelines. Int. J. Surg..

[8] Alexander E.J., White I.M., Horwich A. (2010). Update on management of seminoma. Indian J. Urol..

[9] Naouar Sahbi, Braiek Salem, El Kamel Rafik (2017). Testicular torsion in undescended testis: a persistent challenge. Asian J. Urol..

[10] Ritzen E.M., Bergh A., Bjerknes R. (2007). Nordic consensus on treatment of undescended testes. Acta Paediatr..

[11] Abu-Zaid A., Azzam A., Amin T. (2013). Mixed germ cell tumor complicating an intra-abdominal cryptorchidism. Hematol. Stem Cell Ther..

[12] Sert Ö.Z., Bozkurt H., Senger A.S., Güneş Ö.H. (2019). Mixed germ cell tumor of metastatic undescended testicle causing major GIS bleeding. Urol. Case Rep..

